# Availability, Pharmaceutics, Security, Pharmacokinetics, and Pharmacological Activities of Patchouli Alcohol

**DOI:** 10.1155/2017/4850612

**Published:** 2017-03-21

**Authors:** Guanying Hu, Cheng Peng, Xiaofang Xie, Sanyin Zhang, Xiaoyu Cao

**Affiliations:** ^1^Key Laboratory of Standardization of Chinese Herbal Medicines of the Ministry of Education, Pharmacy College, Chengdu 611173, China; ^2^TCM Qi & Blood Functional Laboratory, College of Basic Medicine, Chengdu University of Traditional Chinese Medicine, Chengdu 611173, China

## Abstract

Patchouli alcohol (PA), a tricyclic sesquiterpene, is one of the critical bioactive ingredients and is mainly isolated from aerial part of* Pogostemon cablin* (known as guanghuoxiang in China) belonging to Labiatae. So far, PA has been widely applied in perfume industries. This review was written with the use of reliable information published between 1974 and 2016 from libraries and electronic researches including NCKI, PubMed, Reaxys, ACS, ScienceDirect, Springer, and Wiley-Blackwell, aiming at presenting comprehensive outline of security, pharmacokinetics, and bioactivities of PA and at further providing a potential guide in exploring the PA and its use in various medical fields. We found that PA maybe was a low toxic drug that was acquired numerously through vegetable oil isolation and chemical synthesis and its stability and low water dissolution were improved in pharmaceutics. It also possessed specific pharmacokinetic characteristics, such as two-compartment open model, first-order kinetic elimination, and certain biometabolism and biotransformation process, and was shown to have multiple biological activities, that is, immunomodulatory, anti-inflammatory, antioxidative, antitumor, antimicrobial, insecticidal, antiatherogenic, antiemetic, whitening, and sedative activity. However, the systematic evaluations of preparation, pharmaceutics, toxicology, pharmacokinetics, and bioactivities underlying molecular mechanisms of action also required further investigation prior to practices of PA in clinic.

## 1. Introduction

**Scheme 1:**
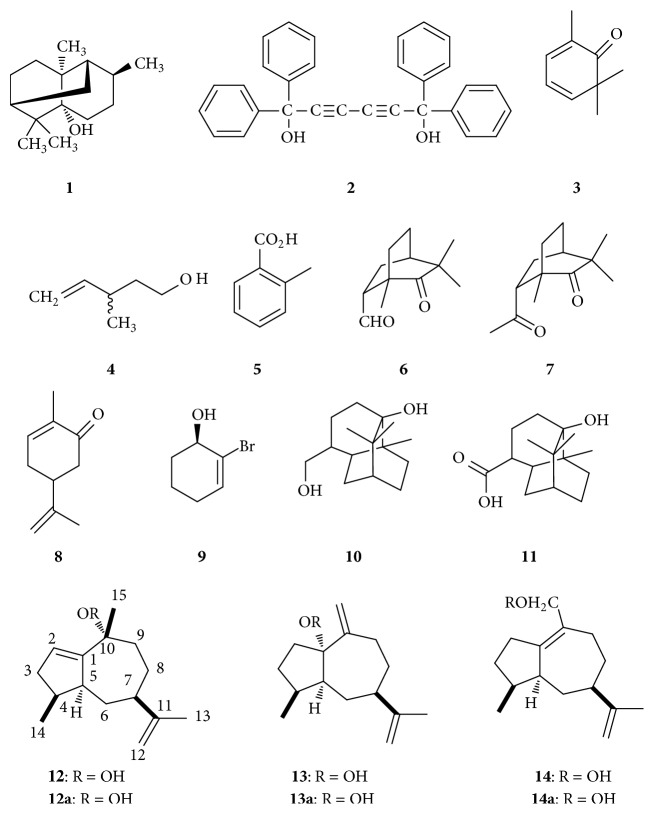


Pogostemonis Herba, the dried aerial part of* Pogostemon cablin* (Blanco) Benth. (Labiatae), is commonly known as “guanghuoxiang” in Chinese and patchouli in English. It was a long time that Pogostemonis Herba had been used traditionally for the treatment of fatigue, summer heat, nausea, vomit, abdominal distension, and so forth [1]. Patchouli oil is an important essential oil in the perfume industry, keeping a base and lasting character to a fragrance. For characteristic pleasant and long lasting woody and camphoraceous odor, patchouli oil is appreciated and very suitable for the utilization of decorative cosmetics, fragrances, shampoo, toilet soaps, and other toiletries as well as noncosmetic products such as household cleaners and detergents [2, 3]. Patchouli alcohol** 1** (PA, C_15_H_26_O), a naturally occurring tricyclic sesquiterpene, is the critically biological active constituent among the patchouli oil extracted from* Pogostemon cablin* and usually used as a pivotal chemical marker compound for the assessments in quality control of Pogostemonis Herba and patchouli oil in China. However, the practices of PA are blank in medical field, although PA has been demonstrated to possess multibeneficial pharmacological properties, such as immunomodulatory, anti-inflammatory, antioxidative, antitumor, antimicrobial, insecticidal, antiatherogenic, antiemetic, whitening, and sedative activities. To provide further supports and evidences for new drug development and clinical application of PA, a comprehensive review on availability, pharmaceutics, security, pharmacokinetics, and pharmacological properties was executed. The abundant and reliable information on the availability, pharmaceutics, safety, pharmacokinetics, and pharmacological properties was collected via libraries and various electronic search engines like CNKI (http://www.cnki.net/), CBM (http://www.sinomed.ac.cn/zh/), PubMed (https://www.ncbi.nlm.nih.gov/pubmed/), Reaxys (http://www.reaxys.com/), ACS (http://pubs.acs.org/), EMBASE (https://www.embase.com/), and HighWire (http://home.highwire.org/). And the important compounds given were listed in Scheme 1.

## 2. Availability

### 2.1. Plant Sources

In nature, PA primarily exists in volatile oil of* Pogostemon cablin* and its contents are about 48.8% [4]. In general, PA contents were higher in the leaves than that in the root and/or stem and altered in different collection parts as well as harvest times. As the determined technology developed, the PA was also detected within volatile oil from other plants, such as Herba* Lysimachia paridiformis* (22.54%) [5], Rhizoma* Valeriana jatamansi jones* (5.88%) [6], Rhizoma* Nardostachys chinensis* (4.5%) [7], Radix* Mallotus apelta* (4.48%) [8], Foliage* Ficus microcarpa *(4.05%) [9], Herba* Pholidota cantonensis *(3.60%) [10], Herba* Asarum sieboldii* (2.75%) [11], Herba* Gendarussa vulgaris* (2.68%) [12], Radix* Helleborus thibetanus* (0.811%) [13], Herba* Sedum sarmentosum* (0.53%) [14],* Aquilaria agallocha *(0.392%) [15], Pericardium* Citri reticulatae* (China: Xinhui, 0.178%; Guangxi, 0.162%; and Fujian, 0.086%) [16], Fructus* Periploca forrestii *Schltr. (MAE, 0.12%; and SDE, 6012%) [17], and Foliage* Microtoena patchouli* [18].

To date, the PA extraction, isolation, and purification from patchouli oil mostly has depended on the combination of silica gel column chromatography and organic solvents participation. On the basis of that, Chen et al. [19] found that using molecular distillation, under the most appropriate conditions (evaporation temperature, 65°C; feeding speed, 120 mL/h; and scraping membrane rate, 150 r/min), raised mass fraction and extraction rate of PA. Qiu et al. [20] observed that employment of supercritical CO_2_ extraction (22 MPa of pressure, 40°C of temperature, and 0.07 mL/min of flow of modifier) optimized by response surface methodology made the yield of PA up to 1.88%. In addition, microwave radiation-accelerated ionic liquid pretreatment (MRAILP) was capable of enhancing extraction of patchouli alcohol (yield, 1.94%) from* Pogostemon cablin* [21]. Furthermore, Li et al. [22] exploited a simple and rapid method for the isolation and purity of PA (yield, 6.28%) by crystalline inclusion of using 1,1,6,6-tetraphenylhexa-2,4-diyne-1,6-diol** 2**. It was worth noting that a rectification-crystallization method was provided for extraction and isolation of PA from patchouli without any organic solvent, and the purity and yield reached 99% and 50%, respectively [23], which was hopefully applied to augment large-scale industrial production.

### 2.2. Chemical Synthesis

Early chemical approach to synthesize PA was through Diels–Alder reaction from the known 2,6,6-trimethyl-2,4-cyclohexadien-l-one** 3** and 3-methylpent-4-en-l-ol** 4** [24]. Magee et al. [25] utilized a 6-exp-trig vinyl radical cyclization methodology to effect six-membered ring closure and then to acquire the desired tricyclic skeleton. Kaliappan and Subba Rao [26] obtained PA by preparing two key intermediates, 6-*endo*-formyl-1,3,3-trimethylbicyclo[2.2.2]octan-2-one** 6** and 6-*endo*-acetyl-1,3,3-trimethylbicyclo[2.2.2]octan-2-one** 7** from 2-methylbenzoic acid** 5**, associated with Birch reduction, Diels–Alder reaction, and catalytic hydrogenation. Moreover, Srikrishna and Satyanarayana [27] synthesized PA from the readily available monoterpene (R)-carvone** 8** through tandem double Michael reaction-alkylation sequence and single electron mediated 6-endo trig cyclization reaction. Recently, the characteristic of allylic substitution of esters derived from 2-bromocyclohex-2-enol** 9** with PhMgBr-based copper reagent has been testified to afford the anti SN2′ products in good yields and with sufficient chirality transfer for synthesis of PA [28].

## 3. Pharmaceutics

Researches made many beneficial tries to improve the oral bioavailability owning to the poor low water solubility of PA in gastrointestinal fluids. PA solid dispersions (SD) formulated with Eudragit were found to result in solution with the highest extent of supersaturation in addition to that the highest concentration of supersaturation of PA was maintained for prolonged time in the PA-SD with Eudragit (E-SD (1/3)) [39]. Liao et al. [40] verified the possibility using poloxamers (188 and 407) as solubility and dissolution rate enhancing agents of PA and suggested that the process of PA-SD pellets preparation was simple, rapid, cost effective, uncomplicated, and potentially scalable. Moreover, there was a communication on improving stability and dissolution rates of PA by complexing with *β*-cyclodextrin, which was confirmed by differential scanning calorimetry (DSC), Fourier transformation-infrared (FT-IR) spectroscopy, powder X-ray diffraction (PXRD), and scanning electron microscope (SEM), respectively [41]. These efforts unambiguously could be an extremely inspiration of PA in pharmaceutical industry and medical practices.

## 4. Security

Acute toxicity investigation, referring to toxic reaction evaluation of animal by single administration or multiply accumulative administration within 24 h, is the first step to assess drug safety. The acute toxicity experiment of PA dissolved by 0.5% CMC-Na in mice was performed early by Ren et al. [42]. After oral and intragastric administration of PA at the maximal dose (0.3115 g/mL) and volume (0.4 mL/10 g) for consecutive 14 d, it gave the maximal tolerance dose of 12.5 g/Kg. Due to the limitation of PA solubility, the LD_50_ values could not be calculated. He et al. [43] also studying acute toxicity of PA which was dissolved with peanut oil in mice concluded LD_50_ values of 4.693 g/Kg and 3.145 g/Kg by intragastric and intraperitoneal injection administration, respectively, and PA thereby was identified to be a low toxic drug. But more preclinical studies, like long-term toxicity, special toxicity, allergy, stimulation, and so forth, on safe assessments of PA were warranted.

## 5. Pharmacokinetics

PA in pharmacokinetics was validated to be in accordance with two-compartment open model and linear kinetics elimination. After intravenous administration of PA (10, 20, and 40 mg·Kg^−1^) to rats, the pharmacokinetic parameters *T*_1/2_*β*, AUC, and MRT examined by capillary gas chromatographic method were 36.8 min, 36534.3 *μ*g·min·L^−1^, and 34.1 min, respectively [44]. Zhang et al. [45] developed a GC-MS assay for the determination of PA in plasma., with the mean AUC_0–t_ of 6916.12 ng·mL^−1^·h^−1^, AUC_0–∞_ of 7896.39 ng·mL^−1^·h^−1^, MRT_0–t_ of 13.93 h, MRT_0–∞_ of 24.33 h, *t*_1/2*z*_ of 19.05, *T*_max_ of 1.04 h, *V*_*z*_/*F* of 36.31 L, Cl_*z*_/*F* of 1.36 L·h^−1^, and *C*_max_ of 545.07 ng·mL^−1^ when the rats were orally gavaged with graded doses of PA (10, 30, and 100 mg·Kg^−1^). Besides, oral administration of PA self-microemulsion (consists of polyoxyethylated castor oil, tween 80, purple glycol 400, isopropyl myristate, and PA at the ratio of 2 : 2 : 0.8 : 1.95 : 0.65, resp.) could remarkably augment AUC values in rats as compared to simple PA [46], suggesting that self-microemulsifying system was in flavor of increasing oral bioavailability of PA. As for metabolism, two hydroxylation metabolites** 10-11** were obtained in liver of rabbits which were treated with PA by intraperitoneal injection [47].

## 6. Pharmacological Activities

### 6.1. Immunomodulatory Activities

Immune system is involved in the etiology and pathophysiological mechanisms of various diseases, and its roles have become more and more important in illuminating the mechanisms for diseases prevention and treatment [48]. Immunomodulation, either by immune increment [49] or by suppression [50], is thought to be an effectively therapeutic strategy to aid in alleviating and even curing diseases [51]. One study indicated that oral administration of PA could enhance phagocytic capability and improve immune organs thymus as well as spleen index and circulating serum IgM and IgG levels. Meanwhile, PA at the dose of 20 mg/Kg significantly repressed delayed type hypersensitivity reaction induced by 2,4-dinitro-chlorobenzene in Kunming mice [52]. In other words, PA exerted appreciated immunomodulatory actions through activating mononuclear phagocytic system, augmenting humoral immune responses, and suppressing cellular immune responses, meaning that PA has a broader prospect used as an immunomodulatory agent served for clinic.

### 6.2. Anti-Inflammatory Activities

In vitro test, the increased productions of TNF-*α*, IL-1*β*, IL-6, PGE_2_, and NO and mRNA expressions of TNF-*α*, IL-1*β*, IL-6, iNOS, and Cox-2 in RAW264.7 cells (a mouse macrophages cell line) stimulated by lipopolysaccharide (LPS) which was endotoxin from Germ negative bacteria were reversed after pretreatment with PA at the concentrations of 10 *μ*M, 20 *μ*M, or 40 *μ*M [53]. Similarly, the in vivo study on anti-inflammation of PA was conducted by Li et al. [54] and oral administration of PA (10, 20, and 40 mg/Kg) could remarkably relieve xylene-induced ear edema in mice as well as carrageenan-induced paw edema in rats, with the reduction of inflammatory mediators release as stated above. Furthermore, PA reflecting clearly anti-inflammatory effects was performed in LPS-induced acute lung injury (ALI) [55, 56] as well as mastitis [57]. The mechanisms were related to the inhibition of phosphorylation of I*κ*B-*α*- and p65-dependent NF-*κ*B transcription activation, thus making it a potential efficiency for the prevention and treatment of inflammatory diseases, like ALI and mastitis. Moreover, Jeong et al. [58] suggested that, in LPS-stimulated RAW264.7 and TNF-*α*-stimulated HT-29 cells (human colorectal adenocarcinoma), PA abated inflammatory responses through suppressing ERK-mediated NF-*κ*B signaling pathway activation.

### 6.3. Antitumor Activities

PA was proved to have marked antitumor abilities against HCT116 and SW480 (human colorectal cancer cells), MCF-7 (human breast cancer cells), BxPC3 (pancreatic cancer cells), and PC3 (human prostate cancer cells), when compared with that acting in HUVEC (human umbilical vein endothelial cells). Also, it was evidenced that PA arrested cell growth and promoted apoptosis in cultured HCT116 and SW480 and thus PA was regarded as cancer specific [59]. In the molecular basis, PA downregulated HDAC2 expression, which consequently attenuated c-myc expression, triggering p21 expression and suppressing cyclin D1 together with CDK4, and activated upon p65-dependent NF-*κ*B transcription. In here, the regulation of NF-*κ*B signaling pathway in response to PA in human colorectal cancer cells was different in TNF-*α*-stimulated HT-29 cells. However, the dual effects of anticancer drug aspirin, similar to PA, also described in other studies, and the mechanisms were attributed to the inhibition of IKK activity and I*κ*B-*α* degradation [60–62]. As for the effects on human androgen independent prostate cancer cells (DU145), Cai et al. observed that PA treatment (10 mg/L to 160 mg/L) not only blocked its cell proliferation detected by MTT but also led to alternation of morphological as well as biochemical features of apoptosis and further elucidated the pathway by which PA induced apoptosis via triggering dissipation of mitochondrial membrane potential after elevating the ratio of Bax/Bcl-2, releasing cytochrome c to the cytosol following the activation of caspase-3, and repressing livin protein expression [63].

### 6.4. Antioxidative Activities

PA was exhibited to have antioxidative activities as follows: (I) PA could block the malondialdehyde (MDA) formation from squalene on UV irradiation by 28.8%, in the thiobarbituric acid assay [64]. (II) As an in vitro study, Liu et al. demonstrated that PA reversed heat stress-stimulated Keap 1 and HO-1 mRNA expressions promotion in cultured IEC-6 (rat intestinal epithelial cell), and the modulatory effects of PA on injured cell by oxidative stress were superior to that of glutamine [65]. (III) Considering that inflammation and oxidative injury interacted with each other, both anti-inflammatory and antioxidative activities of PA were investigated in UV radiation-induced cutaneous photoaging mice [66] and in ethanol, indomethacin, and stress-induced ulcer rats [67]. These results displayed that, except the regulation of inflammatory mediators, oral administration of PA (10, 20, and 40 mg/Kg) also enhanced the activities of SOD, GSH-Px, and CAT, which scavenged ROS by catalyzing them into O_2_ and H_2_O [68], and lowered the contents of MDA, suggesting that PA may be used as a promising reagent served for photoaging and gastric ulcer.

Besides, in the process of screening high throughput drug of estrogen receptor *β* (ER*β*) agonist by established gene report technology, PA was identified to specifically agonize ER*β* which was known to protect nerve from toxicity and to ameliorate animals' learn and cognitive functions. Huang et al. [69] researched the effect of PA on scopolamine-induced memory impairment in mice and found PA treatment for consecutive 10 d could significantly improve the abilities of learn and memory, with the inhibition of AchE activities in the brain, and the elevation of chat activities and M1 receptor levels; in addition to that PA could prevent A*β*_25~35_-induced neuronal apoptotic death through Ca^2+^ load and ROS generation. Thereby, it is possible that PA was explored as novel natural drug against Alzheimer's disease [70].

### 6.5. Antimicrobial Activities

Microbes, also called pathogenic microorganisms, which invade the host, may eventually result in the occurrence of infectious [71] and allergic diseases [72], even the death [73]. At present, the abuse of antibiotics and chemotherapeutic agents has made the pathogens fail to respond to treatment and show drug resistance. Because of no obvious side effects, natural products have received more attentions as antipathogenic microorganism agents for the treatment to drug-resistant pathogenic microorganisms in recent years. PA, one of nature compounds, exhibited directly inhibitory or zapped effects on various pathogens including bacteria, viruses, and fungus, which was listed in Table 1, numbers 1–16.

#### 6.5.1. Antibacterial Activities

The antibacterial activities of patchouli oil were studied by using feasible molecular docking technology and in vitro antibacterial test [29], and the study revealed that, among the 31 chemical compounds, PA was confirmed to be the main principle of patchouli oil in exerting strongly antibacterial potential against* E. coli*,* P. aeruginosa*,* B. proteus*,* S. dysenteriae*,* T. bacillus*, and* S. aureus*. Additionally, PA showed selective antibacterial activities against in vitro* H. pylori* without affecting the normal flora of gastrointestinal tract, and its anti-*H. pylori* effect was greater than that of amoxicillin with MIC value of 78 *μ*g/mL and 120 *μ*g/mL, respectively [30]. Due to its potently inhibitory abilities to urease, thereby, PA can be regarded as a promising agent to cure specific* H. pylori *infection.

#### 6.5.2. Antiviral Activities

Concerning the assessment of in vitro anti-influenza virus (IFV) A/PR/8/34 (H1N1), it was showed that PA, at the concentrations of 2 *μ*g/L and 10 *μ*g/L, reduced the population of plaque by 75% and 89%, respectively, with IC_50_ of 2.635 *μ*M [31]. Similarly, the results presented by Wu et al. [32] were proved to be that anti-IFV strain A/Leningrad/134/17/1957 (H2N2) of PA with IC_50_ of 4.03 *μ*M and suggested that the support of PA used in H2N2-elicited infection was related to interference of PA with in silico NA activities through stabilization by binding NA invariant key active-site residues Asp151, Arg152, Glu119, Glu276, and Tyr406. The in vivo anti-IFV activity of PA was studied as well and the study disclosed oral administration of PA (20 mg/Kg to 80 mg/Kg) would be able to augment protection against influenza viral infection mice by increasing CD3+ and CD4+ T cell levels as well as the CD4+/CD8+ ratio, and anti-IFV IgA, IgM, and IgG titers productions to enhance the host immune responses and by restraining TNF-*α*, IL-4, and IFN-*γ* inflammatory factors release to attenuate systemic and pulmonary inflammatory responses [33]. Moreover, Wu et al. [34] investigated the interference of PA in infectious 16HBE (human respiratory epithelial cell) by A/FM1/1/47 (H1N1) and found PA could evoke the abilities of natural immune recognition and responses but attenuate the inflammatory responses through repressing the IFN-*γ* expression.

#### 6.5.3. Antifungal Activities

The mediate (43.62 ppm) and highest concentrations (65.42 ppm) of PA were showed to have antifungal activities against all tested fungi, including* A. flavus* (3.2758),* A. flavus* (3.4408), and* A. oryzae*, at either low (0.95) or high (0.98) *a*_*w*_ (water activity) level, via reducing colony growth rate, which represented a available solution for possible application of PA in the food industry [35]. Besides, Mirko et al. [74] found that PA could inhibit the asexual propagation of fungi and prevent the adhesion of microorganisms to surfaces, and therefore PA was recommended to be used in life household, like filter media, adhesives, building materials, building auxiliaries, laundry detergents, cleaning compositions, rinse agents, fabric treatment compositions, hand washing compositions, manual dishwashing detergents, machine dishwashing detergents, cosmetic compositions, pharmaceutical compositions, oral hygiene compositions, dental care compositions, and denture care compositions.

### 6.6. Insecticidal Activities

The experiment on repellent and toxic effects of PA on* Coptotermes formosanus* Shiraki (Isoptera: Rhinotermitidae) was carried out by Zhu et al. [36]. The results uncovered that PA pretreatment resulted in elevation in mortality percentage and decline in termites feeding, contacting and tunneling behaviors, and particularly the internal tissue of termites was destructed inside the exoskeleton when PA was topically applied into the dorsum (Table 1, number 17). Furthermore, PA was witnessed to have obvious repellency and toxicity towards mosquitoes (Table 1, numbers 18–20). PA, at 2 mg/cm^2^ concentration, was the most effective for repellent activity, providing 100% protection up to 280 min, against* Ae aegypti*,* An. Stephensi,* and* Cx. quinquefasciatus*, and for pupicidal activity at 100 mg/L concentration, providing 28.44, 26.28, and 25.36 against above vector mosquitoes tested [37]. These findings provide experimental basis for the development of PA as an ideal eco-friendly pesticide for the control of termites and mosquitoes.

There was another published paper where three new sesquiterpene hydroperoxides** 12–14** isolated from acetone extract of Pogostemonis Herba exhibited potent trypanocidal activities, and the MLC values were estimated to be 0.84 *μ*M, 1.7 *μ*M, and 1.7 *μ*M, respectively, when compared with that of a known sesquiterpene patchouli alcohol (MLC > 200 *μ*M) (Table 1, number 21). It suggests that it is operational to improve the trypanocidal actions by appropriate structural modification of PA [38].

### 6.7. Antiatherogenic Activities

The study using atherosclerosis-prone apolipoprotein E knockout mice was carried out by Wang et al. [75] who demonstrated PA had antiatherogenic actions through attenuating atherosclerotic plaque burdens in both the aorta and the aortic root, reducing macrophage infiltration, and repressing inflammatory response via downregulation of MCP-1, iNOS, IL-1b, IL-6, CXCL9, and CXCL11 expressions.

### 6.8. Antiemetic Activities

Yang et al. [76] observed that treatment of young chickens with 50 mg/Kg and 70 mg/Kg of PA lowered excessive contractions of digestive organ smooth muscles by reducing extracellular Ca^2+^ influx, verifying PA in Pogostemonis Herba may play a pivotal role in antiemetic effects clinically in view of utilization of TCM.

### 6.9. Whitening Activities

In B16 melanoma cells, PA treatment could inhibit melanin synthesis in a dose-dependent manner (IC_50_ = 3.9 *μ*g/mL), weaken ROS scavenging activities in DPPH radical (IC_50_ = 3.14 ± 0.12 *μ*g/mL) and xanthine/xanthine oxidase system (IC_50_ = 49 ± 3.24 *μ*g/mL) and intracellular tyrosinase activities, and suppress tyrosinase as well as TRP-2 expressions [77], suggesting that PA could be used as a useful whitening agent.

### 6.10. Sedative Activities

It was reported that single inhalation administration of PA which stemmed from* Microtoena patchouli* presented clearly sedative effects by suppressing spontaneous motor activities [18].

## 7. Conclusions

PA is being explored and used in fragrance industries, but there is currently no report on application of PA in medical fields. This review summarizes the updated researches published on the availability, pharmaceutics, security, pharmacokinetics, and pharmacological activities of PA, which may aid in accelerating its development and medical practices, but what it chiefly needs to overcome is high cost industrial production and poor low water dissolution of PA.

**Figure 1:**
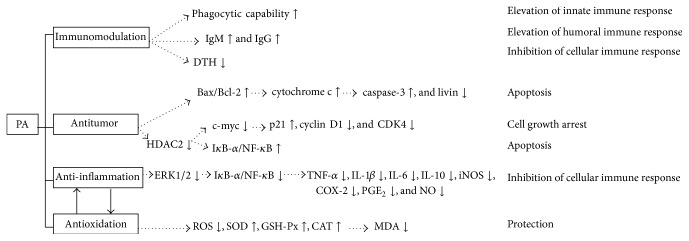
Possible mechanisms of bioactivities of PA in immunomodulation, antitumor, anti-inflammation, and antioxidation. Arrow up denotes activation or increase; arrow down denotes suppression or decrease. Inflammation and oxidation interact with each other.

The present information exhibited remarkably therapeutic properties of PA in vivo and in vitro, which could be contributed to the prevention and treatment of many diseases, such as immune disorders, infections by microbes, mosquitoes or* Trypanosoma cruzi*, ALI, mastitis, gastric ulcer, skin photoaging, atherosclerosis, and tumor. As shown in Figure 1, PA totally had powerful bioactivities to regulate immune functions, including immune defense, homeostasis, and surveillance. However, further studies are required for investigating PA which especially is how to accurately and bilaterally regulate the NF-*κ*B nuclear transcription and the release of inflammatory factors.

Although various bioactivities of PA were authenticated using laboratory animals and cells, few molecular mechanisms of action and definite target protein bound by PA are known, in addition to limited efforts made in both the pharmacokinetic investigation related to the mechanisms of action and toxicological assessments of PA, which may severely hamper development of new drug and clinical applications of PA.

## Figures and Tables

**Table 1:** The list of antimicrobial and insecticidal activity of PA.

Numbers	Tested mode	Tested subject	Parameters (MIC/IC_50_/LD_50_/MLC)	References
Bacteria				

1	In vitro	*E. coli*	MIC = 1.0 mg/mL	[29]
2	In vitro	*P. aeruginosa*	MIC = 3.5 mg/mL	[29]
3	In vitro	*B. proteus*	MIC = 3.5 mg/mL	[29]
4	In vitro	*S. dysenteriae*	MIC = 3.0 mg/mL	[29]
5	In vitro	*T. bacillus*	MIC = 6.5 mg/mL	[29]
6	In vitro	*S. aureus*	MIC = 2.0 mg/mL	[29]
7	In vitro	*H. pylori*	MIC = 78 *μ*g/mL	[30]

Viruses				

8	In vitro	A/PR/8/34 (H1N1)	IC_50_ = 2.635 *μ*M	[31]
9	In vitro	B/Ibaraki/2/85	IC_50_ = 40.82 *μ*M	[31]
10	In vivo	A/Leningrad/134/17/1957 (H2N2) infection mice	—	[32]
11	In vitro	A/Leningrad/134/17/1957 (H2N2)	IC_50_ = 4.03 *μ*M	[32]
12	In vivo	A/FM1/1/47 (H1N1) infection mice	—	[33]
13	In vitro	A/FM1/1/47 (H1N1) infection 16HBE	—	[34]

Fungus				

14	In vitro	*A. flavus* (3.2758)	—	[35]
15	In vitro	*A. flavus* (3.4408)	—	[35]
16	In vitro	*Aspergillus oryzae*	—	[35]

Termite				

17	In vivo	*Coptotermes formosanus* Shiraki	LD_50_ = 4.57 *μ*g/termite	[36]

Mosquitoes				

18	In vivo	*Ae. aegypti*	—	[37]
19	In vivo	*An. stephensi*	—	[37]
20	In vivo	*Cx. quinquefasciatus*	—	[37]

*Trypanosoma*				

21	In vivo	*Trypanosoma cruzi*	MLC > 200 *μ*M	[38]

MIC, minimal inhibition concentration; IC_50_, 50% of lethal concentration; LD_50_, 50% of lethal dose; MLC, minimal lethal concentration.
